# The Trajectory Patterns of Parenting and the Social Competence of Toddlers: A Longitudinal Perspective

**DOI:** 10.2188/jea.JE20090172

**Published:** 2010-03-05

**Authors:** Yuka Sugisawa, Ryoji Shinohara, Lian Tong, Emiko Tanaka, Taeko Watanabe, Yoko Onda, Yuri Kawashima, Maki Hirano, Etsuko Tomisaki, Yukiko Mochizuki, Kentaro Morita, Amarsanaa Gan-Yadam, Yuko Yato, Noriko Yamakawa, Tokie Anme, Japan Children’s Study Group

**Affiliations:** 1 Research Institute of Science and Technology for Society, Japan Science and Technology Agency, Tokyo, Japan; 2 Graduate School of Comprehensive Human Sciences, University of Tsukuba, Tsukuba, Ibaraki, Japan; 3 College of Letters, Ritsumeikan University, Kyoto, Japan; 4 Clinical Research Institute, Mie-Chuo Medical Center National Hospital Organization, Tsu, Japan

**Keywords:** mother–child interaction, social competence, child care, child development, Japan

## Abstract

**Background:**

Many studies have suggested that the daily emotional interactions between a child and his/her caregiver play a significant role in his/her development. The purpose of this study was to determine whether the trajectory patterns of parenting patterns of caregivers raising toddlers affect the social competence of the toddlers.

**Methods:**

The study participants were 246 dyads of 18-month-old children (baseline) and their caregivers, which was conducted as part of a Japan Science and Technology Agency (JST) project. We used the Interaction Rating Scale (IRS) to evaluate the children’s social competence. We assessed the child rearing environments by analyzing the caregivers’ responses to the Index of Child Care Environment (ICCE).

**Results:**

Multiple logistic regression analysis showed that the children’s total score on the IRS was significantly related to how frequently they sang songs together with their caregivers. Their score was also significantly related to how closely their caregiver worked with his/her partner in raising the child. These relationships did not change according to demographic information.

**Conclusions:**

The results confirm previous findings on the relationship between parenting patterns and children’s social competence. In particular, the study shows that varied and continual parenting significantly affects a child’s social competence.

## INTRODUCTION

Children’s level of social competence is an outcome of complex interactions.^1^ Indeed, a number of studies have attempted to determine the importance of children’s rearing environment to the development of their social competence.

There exists in-depth research on the relationship between parenting patterns and children’s social competence.^2^ In recent years, the focus of the research has shifted from parenting patterns such as discipline to family-peer connections, including the relationship between a child’s social competence and the social-emotional environment at home or the manner in which the caregiver expresses his/her feelings to the child while playing.^3^ These researches suggest that the daily emotional interactions between a child and his/her caregiver play a significant role in his/her development.

Most of the above research has focused on children between kindergarten-age and adolescence because the range of social competence increases as children begin to interact in peer relationships and their level of social competence assumes increasing importance in their daily interactions. However, behavioral research on the daily interactions between a child and his/her caregiver has not yielded any definitive conclusions on how such interactions affect children’s social competence.

We conduct a longitudinal study to assess young children’s rearing environment. The purpose of the study is to examine the effect of parenting at 18- and 30–month- on the social competence of 30-month-olds.

## METHODS

### Participants

The research was conducted as a one-year longitudinal prospective cohort study in 2007–2008. The participants were 246 dyads of children (boys: 125; girls: 121) aged 18 months (baseline) and their caregivers. Because this study is part of a Japan Science and Technology Agency (JST) project, the participants were selected from among those who had participated in the project; we excluded those for whom we could not obtain complete demographic information or data for the past 30 months. The demographic information we examined included the following: gender of child, number of siblings, family type, mother’s age, mother’s education, mother’s occupation, father’s age, father’s education, and economic status of the family.

We complied with the ethical standards laid down by the JST. The families of all the participants signed informed consent forms before the experiment began, and they were made aware of their right to withdraw from the experiment at any time. Because the toddlers were too young to provide informed consent, we carefully explained the purpose, content, and methods of the study to the caregivers and obtained their consent on behalf of the toddlers. To maintain confidentiality, the participants’ personal information was collected anonymously, and a personal ID system was employed when the information was used. Further, all image data were stored on a password-protected disk. Finally, researchers were required to obtain permission from the chairman to access the data.

This study was approved by the ethics committee of the JST.

### Measures

#### Interaction Rating Scale

*Description.* We used the interaction rating scale (IRS) to evaluate the children’s social competence on the basis of parent-child interactions. The reliability and validity of the IRS have been confirmed.^4^

The IRS has 10 subscales: 5 related to the child and 5 pertaining to the caregiver. Since we are only concerned with the children’s social competence, we used the subscales relating to the child, which are as follows: (1) autonomy, (2) responsiveness, (3) empathy, (4) motor self-regulation, and (5) emotional self-regulation. Each of these subscales further consists of 5 items. For example, the autonomy subscale includes the item “child attempts to make eye contact with caregiver”; the responsiveness subscale, “child vocalizes or babbles within 5 seconds after caregiver’s verbalization”; the empathy subscale, “child gives, shows, or points to task material to share emotion with caregiver”; the motor self-regulation, “child makes clearly recognizable hand motions towards task materials during the episode (60% or more of the time)”; and the emotional self-regulation subscale, “child stops displaying distress cues within 15 seconds of caregiver’s soothing attempts.”

*Observations.* The observation was carried out in a controlled laboratory environment. The child and caregiver interacted by playing with blocks and putting them in a box. At the start of the experiment, the child-caregiver dyad was escorted into a room (4 × 4 meters in size) furnished with a small table and a child-sized chair. The caregiver would introduce herself to the child and interact with the child in a natural manner, just as she would on a regular day. The interactions were videotaped for 5–10 minutes using 5 video cameras (four were placed at each corner of the room and one was placed in the central ceiling position).

*Scoring.* Two members of the research team coded the children’s behaviors. They are a child specialist and who had no contact with the participants. The behavior of the children and caregivers during the caregiver-child interactions was coded as follows. If the child displayed the behavior described in the IRS item, a score of 1 was given; conversely, if the child failed to display the behavior described in the item, a score of 0 was given. A child’s total score was the sum of the score that he/she received on all the subscales. A high score indicated a high level of development. The 10th percentile of this data set (measured from the negative region of the spectrum) was used as the cut-off point to determine the high scoring (18–25 points) and low scoring (0–17 points) groups (see Table 1).

**Table 1.** Demographic information and children’s scores on the IRS

Items		categries	Total Score on the IRS (0–25 points)		High Scoring Group (18–25 points)		Low Scoring Group (0–17 points)	
		
			*n*	%	*n*	%	*n*	%
Gender	Boys	1	125	50.8	107	48.4	18	72.0
	Girls	2	121	49.2	114	51.6	7	28.0

Siblings	No	1	137	55.7	121	54.8	16	64.0
	Yes	2	109	44.3	100	45.3	9	36.0

Family type	Nuclear family	1	217	88.2	195	88.2	22	88.0
	Extended family	2	29	11.8	26	11.8	3	12.0

Mother’s age	20–29	1	60	24.4	56	25.3	4	16.0
	30–39	2	174	70.7	156	70.6	18	72.0
	40–	3	12	4.9	9	4.1	3	12.0

Mother’s education	Intermediate school	1	3	1.2	3	1.4	0	0.0
	High school	2	49	19.9	45	20.4	4	16.0
	Technical college	3	50	20.3	43	19.5	7	28.0
	Junior college	4	71	28.9	68	30.8	3	12.0
	University	5	72	29.3	61	27.6	11	44.0
	Graduate	6	1	0.4	1	0.5	0	0.0

Mother’s occupation	No	1	123	50.0	112	50.7	11	44.0
	Yes	2	123	50.0	109	49.3	14	56.0

Father’s age	20–29	1	46	18.7	43	19.5	3	12.0
	30–39	2	169	68.7	152	68.8	17	68.0
	40–49	3	27	11.0	23	10.4	4	16.0
	50–	4	4	1.6	3	1.4	1	4.0

Father’s education	Intermediate school	1	4	1.6	4	1.8	0	0.0
	High school	2	81	32.9	71	32.1	10	40.0
	Technical college	3	36	14.6	33	14.9	3	12.0
	Junior college	4	6	2.4	6	2.7	0	0.0
	University	5	104	42.3	93	42.1	11	44.0
	Graduate	6	15	6.1	14	6.3	1	4.0

Family’s economic status ​ (annual income)	<2 million JPY	1	7	2.9	5	2.3	2	8.0
	2–4 million JPY	2	60	24.4	53	24.0	7	28.0
	4–6 million JPY	3	120	48.8	109	49.3	11	44.0
	6–8 million JPY	4	34	13.8	30	13.6	4	16.0
	8–10 million JPY	5	14	5.7	13	5.9	1	4.0
	≧10 million JPY	6	11	4.5	11	5.0	0	0.0

*N* = 246.

#### Index of Child Care Environment

*Description.* The Index of Child Care Environment (ICCE)—which is based on the Home Observation for Measurement of the Environment^5^—is a screening questionnaire used to evaluate the quality of the child care environment. The reliability and validity of the ICCE have been confirmed.^6^^–^^8^ The ICCE contains 13 items clustered into 4 subscales: (1) human stimulation, (2) avoidance of restriction, (3) social stimulation, and (4) social support. In this study, we only used the positive subscales; therefore, we excluded the avoidance of restriction subscale. The items included in the 3 subscales are as follows (the labels in parentheses indicate their description in the tables). The human stimulation subscale includes “how often do you play with your child per week?” (play with your child), “how often do you read to your child?” (read books to your child), “how often do you sing songs with your child?” (sing songs with your child), “how often does your spouse, partner, or other care giver help you?” (work together with your partner to raise your child), “how often does your child eat meals together with both parents?” (eat meals together as a family). The social stimulation subscale includes “how often do you go shopping with your child?” (go grocery shopping with your child), and “how often do you go to the park with your child?” (go to the park with your child), “how often do you and your child meet with friends or relatives with children of a similar age?” (go to friends’ or relatives’ house). Finally, the social support subscale includes “how many times do you have a chance to talk with your partner about your child?” (talk with your partner about your child), “does someone help you take care of your child?” (have child care support), “do you have someone to consult with about child care?” (have consult).

*Coding of the Responses.* Caregivers completed the ICCE twice, first when their child was 18 months old and then when their child was 30 months old. Some of the items were rated on a five-point scale (from 1 to 5); others required a simple yes-or-no response.

The responses to the items in the questionnaire were coded as “Recoded Variable I”: Most positive responses were coded as 1, and the others were coded as 0 (see Table 2). Next, consistency between the responses was coded as “Recoded Variable II” in the following manner (see Table 3):

if Recoded variable I = (0, 0) (18 months, 30 months), then Recoded variable II = 0 (consistently negative group);
if Recoded variable I = (1, 0), then Recoded variable II = 1 (inconsistent group);
if Recoded variable I = (0, 1) then Recoded variable II = 1 (inconsistent group); and
if Recoded variable I = (1, 1) then Recoded variable II = 2 (consistently positive group).

**Table 2.** Responses on the ICCE (Recoded Variable I)

Items	Categories	Recoded Variable I	18M		30M	
	
			*n*	%	*n*	%
*Human stimulation*						
Play with your child	1. very infrequently	0	27	11.0	49	19.9
	2. 1–2 times/week					
	3. 3–4 times/week					
	4. 5–6/week					
	5. almost every day	1	219	89.0	197	80.1

Read books to your child	1. very infrequently	0	156	63.4	164	66.7
	2. 1–2 times/month					
	3. 1–2 times/week					
	4. 3–4 times/week					
	5. almost every day	1	90	36.6	82	33.3

Sing songs with your child	1. very infrequently	0	93	37.8	67	27.2
	2. 1–2 times/month					
	3. 1–2 times/week					
	4. 3–4 times/week					
	5. almost every day	1	153	62.2	179	72.8

Work together with your partner to raise	1. very infrequently	0	95	38.6	108	43.9
your child	2. 1–2 times/month					
	3. 1–2 times/week					
	4. 3–4 times/week					
	5. almost every day	1	151	61.4	138	56.1

Eat meals together as a family	1. very infrequently	0	59	24.0	71	28.9
	2. 1–2 times/month					
	3. 1–2 times/week					
	4. 3–4 times/week					
	5. almost every day	1	187	76.0	175	71.1

*Social stimulation*						
Go grocery shopping with your child	1. very infrequently	0	171	69.5	177	72.0
	2. 1–2 times/month					
	3. 1–2 times/week					
	4. 3–4 times/week					
	5. almost every day	1	75	30.5	69	28.0

Go to the park with your child	1. very infrequently	0	169	68.7	190	77.2
	2. 1–2 times/month					
	3. 1–2 times/week					
	4. 3–4 times/week					
	5. almost every day	1	77	31.3	56	22.8

Go to friends’ or relatives’ house	1. very infrequently	0	239	97.2	231	93.9
	2. 1–2 times/month					
	3. 1–2 times/week					
	4. 3–4 times/week					
	5. almost every day	1	7	2.8	15	6.1

*Social support*						
Talk with your partner about your child	1. very infrequently	0	72	29.3	85	34.5
	2. 1–2 times/month					
	3. 1–2 times/week					
	4. 3–4 times/week					
	5. almost every day	1	174	70.7	161	65.5

Have child care support	1. No	0	15	6.1	14	5.7
	2. Yes	1	231	93.9	232	94.3

Have consult	1. No	0	1	0.4	2	0.8
	2. Yes	1	245	99.6	244	99.2

**Table 3.** Single logistic regression analysis between IRS scores and ICCE responses (Recoded Variable II)

Items (Change)	Recoded Variable I		Recoded Variable II	*n*	%	OR	CI	*P*

	18M	30M						
*Human stimulation*								
Play with your child	0	0	0	15	6.1	0.91	0.43–1.90	0.7922
	1	0	1	46	18.7			
	0	1						
	1	1	2	185	75.2			

Reading books to your child	0	0	0	130	52.9	1.11	0.66–1.86	0.7022
	1	0	1	60	24.4			
	0	1						
	1	1	2	56	22.8			

Sing songs with your child	0	0	0	46	18.7	1.82^a^	1.10–3.01	0.0204
	1	0	1	68	27.6			
	0	1						
	1	1	2	132	53.7			

Work together with your partner to raise	0	0	0	71	28.9	1.66^a^	1.02–2.71	0.0416
your child	1	0	1	61	24.8			
	0	1						
	1	1	2	114	46.3			

Eat meals together as a family	0	0	0	35	14.2	1.44	0.85–2.43	0.1709
	1	0	1	60	24.4			
	0	1						
	1	1	2	151	61.4			

*Social stimulation*								
Go grocery shopping with your child	0	0	0	144	58.5	1.05	0.61–1.82	0.8610
	1	0	1	60	24.4			
	0	1						
	1	1	2	42	17.1			

Go to the park with your child	0	0	0	157	63.8	1.33	0.74–2.40	0.3441
	1	0	1	45	18.3			
	0	1						
	1	1	2	44	17.9			

Go to friends’ or relatives’ house	0	0	0	225	91.5	1.13	0.27–4.83	0.8681
	1	0	1	20	8.1			
	0	1						
	1	1	2	1	0.4			

*Social support*								
Talk with your partner about your child	0	0	0	45	18.3	1.00	0.59–1.71	0.9902
	1	0	1	67	27.2			
	0	1						
	1	1	2	134	54.5			

Have child care support	0	0	0	5	2.0	0.71	0.19–2.62	0.6024
	1	0	1	19	7.7			
	0	1						
	1	1	2	222	90.2			

Have consult	0	0	0	1	0.4	—	—	—
	1	0	1	1	0.4			
	0	1						
	1	1	2	244	99.2			

Because there is one person in each of Recoded Variable II ‘0’ and ‘1’ of ‘Have consult’.

^a^*P* < 0.05.

#### Statistical Analysis

Single logistical regression analysis was used to examine the relationship between the children’s total score on the IRS (high and low scoring groups) and Recoded variable II as computed from the ICCE (consistently negative group, inconsistent group, consistently positive group). Only the factors which met the statistical significance level in the single logistical regression analysis were put into the multiple model with demographic factors. The statistical significance level was 5%.

The analysis was performed using the software package Statistical Analysis System (SAS, Ver. 9.1).

## RESULTS

Demographic data pertaining to the participants are shown in Table 1. The children’s group was composed of an almost equal proportion of boys (125, 50.8%) and girls (121, 49.2%). A little more than half the children were a single child, and most of them lived in a nuclear family. The majority of participating mothers were between 30 and 39 years of age (174, 70.7%), as were most of the fathers (169, 68.7%). Boys were more likely to have low scores on the IRS, with more girls than boys in the high scoring group (boys; 48.4%, girls; 51.6%), and a large number of boys in the low scoring group (boys: 72.0%, girls: 28.0%).

Table 2 shows the responses provided on the ICCE. In the domain of human stimulation, caregivers indicated that they played with their child (18M: 89.0%, 30M: 80.1%) and read books to their child (18M: 36.6%, 30M: 33.3%) almost every day. Further, 60% and 70% (at 18M and 30M respectively) indicated that they sang songs with their child, worked together with their partner to raise their child, and ate meals together as a family almost every day. In the social stimulation domain, 20–30% of the caregivers answered that almost every day, they took their child with them when they went grocery shopping and took their child to the park. However, less than 10% responded that they took their child to a friend’s or relative’s house. In the social support domain, caregivers indicated that they talked to their partner about their child almost every day (18M; 70.7%, 30M; 65.5%). Finally, more than 90% answered that they received child care support and consulted with someone to discuss child care.

Table 3 presents the results of the single logistic regression analysis, which was performed to determine the relationship between a child’s social competence (children’s total scores on the IRS) and child care environment (the ICCE scores). The analysis showed that the children’s total score on the IRS was significantly related to “sing songs with your child” (OR = 1.82, CI: 1.10–3.01), and “work together with your partner to raise your child” (OR = 1.66, CI: 1.02–2.71). This indicated that, for example, children who often sang a song with their caregivers when they were 18 months old and 30 months old developed a higher level of social competence at the latter age, with an odds ratio of 1.82, than children who did not sing songs with their caregivers.

Multiple logistic regression analysis was performed to determine the relationships between the items the children’s total scores on the IRS and “sing songs with your child”, and the children’s total scores on the IRS and “work together with your partner to raise your child”; demographic information was controlled for in this analysis (Tables 4 and 5).

**Table 4.** Single and multiple logistic regression analyses (for item “sing songs with your child”)

Items	single			multiple		
	
	OR	95% CI	*P*	OR	95% CI	*P*
**Sing songs with your child**	1.82^a^	1.10–3.01	0.0204	1.71^a^	1.01–2.89	0.0475
Gender	2.74^a^	1.10–6.82	0.0303	2.73^a^	1.04–7.18	0.0416
Siblings	1.47	0.62–3.47	0.3799	1.70	0.68–4.22	0.2536
Family type	0.98	0.27–3.49	0.9722	0.89	0.22–3.70	0.8735
Mother’s age	0.49	0.21–1.17	0.1067	0.60	0.20–1.81	0.3631
Mother’s education	0.86	0.59–1.24	0.4141	0.85	0.56–1.29	0.4560
Mother’s occupation	0.77	0.33–1.76	0.5276	0.75	0.31–1.84	0.5340
Father’s age	0.62	0.32–1.19	0.1490	0.68	0.27–1.71	0.4093
Father’s education	1.05	0.79–1.39	0.7451	0.97	0.68–1.38	0.8496
Family’s economic status	1.38	0.88–2.15	0.1600	1.50	0.99–2.52	0.1239

H-L test (*P*)	—			0.3878		

Recoded Variable II is used in this analysis.

^a^*P* < 0.05.

**Table 5.** Single and multiple logistic regression analysis (for item “work with your partner to raise your child”)

Items	single			multiple		
	
	OR	95% CI	*P*	OR	95% CI	*P*
**Work with your partner to raise your child**	1.66^a^	1.02–2.70	0.0416	1.79^a^	1.06–3.02	0.0286
Gender	2.74^a^	1.10–6.82	0.0303	3.24^a^	1.21–8.65	0.0192
Siblings	1.47	0.62–3.47	0.3799	1.54	0.61–3.87	0.3643
Family type	0.98	0.27–3.49	0.9722	0.78	0.18–3.34	0.7357
Mother’s age	0.49	0.21–1.17	0.1067	0.60	0.19–1.88	0.3798
Mother’s education	0.86	0.59–1.24	0.4141	0.82	0.54–1.24	0.3543
Mother’s occupation	0.77	0.33–1.76	0.5276	0.67	0.27–1.63	0.3736
Father’s age	0.62	0.32–1.19	0.1490	0.70	0.28–1.79	0.4586
Father’s education	1.05	0.79–1.39	0.7451	1.03	0.72–1.48	0.8658
Family’s economic status	1.38	0.88–2.15	0.1600	1.51	0.99–2.53	0.1202

H-L test (*P*)	—			0.6133		

Recoded Variable II is used in this analysis.

^a^*P* < 0.05.

The results showed that the children’s total score on the IRS was significantly related to they sang songs with their caregivers (OR = 1.71, CI: 1.01–2.89), and their caregivers worked together with their partner to raise their child (OR = 1.79, CI: 1.06–3.02) even when demographic information was controlled.

## DISCUSSION

This study found that a strong relationship exists between the environment in which a child is raised and his/her social competence. For example, we found that children who often sang songs with their caregivers when they were 18 months old and 30 months old had developed a higher level of social competence at the latter age than those who did not often sing with their caregivers. The same result was found in the case where the caregivers worked together with their partner to raise their child.

The results pertaining to the item “sing songs with your child” suggest that, for example, when a child and his/her caregiver spend time together by singing songs, a positive emotional interaction takes place between them. This result supports recent findings that the social-emotional environment at home or the manners in which caregivers express their feelings to their child while playing is related to children’s social competence.^3^^,^^9^

There are two possible implications of the results pertaining to the item “worked together with your partner to raise your child.” First, active involvement of the caregiver’s partner leads to more human stimulations for the child than in the case where only one caregiver raises the child. Therefore, this factor affects children’s social competence. Second, because the involvement of the partner helps reduce the child care burden of the caregiver, the caregiver is generally under less stress as compared to those who do not receive such support. This indirectly affects the relationship between the caregiver and child.

For more than 30 years, Lamb and his research group have been accumulating data and knowledge on the affects of both fathers’ and mothers’ parenting styles on children’s social competence.^10^^,^^11^ Other recent studies have found relations between fathers’ parenting patterns or father-child interactions and child development.^12^^,^^13^ These researches clearly reveal the significant quantitative and qualitative effects of a father’s relationship with his child on the child’s social and cognitive development.^14^ Our results confirm these findings and indicate the important contribution that continual interactions with both mother and father make to children’s social development. The study further finds that daily, positive social-emotional interactions between children and caregivers, such as often singing songs together, possibly affect children’s social competence.

Although the present study provides some valuable insights into the factors that affect children’s social competence, it is important to acknowledge its limitations. First, although the study had used a two-stage longitudinal design and the children’s child-rearing environment was assessed when they were 18 months old and 30 months old, a three-stage longitudinal design could better clarify the effects of different child rearing environments. Second, a study with a larger sample group is required to ascertain the effects of demographic information: especially children’s gender on children’s social development.

Despite the limitations, we are confident that our results sufficiently establish that parenting patterns and activities—particularly singing songs with one’s child and working together with one’s partner to raise one’s child—are associated with the social competence of 30-month-olds. This study underscores the importance of varied and continual parenting when the child is very young.

We are currently in the process of analyzing data pertaining to 42-month-olds. We believe that our research could reveal various aspects of children’s social competence, which can have important implications for caregivers and child care professionals.
